# *Pasteurella multocida* Bacteremia in an Immunocompromised Patient

**DOI:** 10.1155/2016/7392847

**Published:** 2016-10-25

**Authors:** Shweta Kukrety, Jai Parekh, Theresa Townley

**Affiliations:** Department of Internal Medicine, Creighton University Medical Center, Omaha, NE, USA

## Abstract

We present the case of a 61-year-old Caucasian gentleman who presented with a one-day history of fever, chills, and altered mental status. His symptoms were initially thought to be secondary to cellulitis. Blood cultures grew* Pasteurella multocida*, a rare pathogen to cause bacteremia. Our patient was treated with ciprofloxacin for two weeks and made a complete and uneventful recovery. Our patient's uncontrolled diabetes mellitus and chronic kidney disease put him at a higher risk for developing serious* P. multocida* infection. The patient's dog licking the wounds on his legs was considered as the possible source of infection. As* P. multicoda* bacteremia is rare, but severe with a high mortality rate, it is imperative to have a high index of suspicion for this infection especially in the vulnerable immunocompromised population.

## 1. Introduction

*Pasteurella multocida* was named after Louis Pasteur who first described the organism as a causative agent of fowl cholera in 1881 [1].* P. multocida* are normal commensals in the oropharynx of domestic animals like cats and dogs. The majority of* P. multocida* infections involve skin and soft tissue and complicate a bite or scratch. Rarely it results in a more serious systemic illness, especially in patients with chronic predisposing conditions. In this article we describe the epidemiology, clinical features, treatment, and prognosis of* P. multocida* infections.

## 2. Case Presentation

A 61-year-old Caucasian gentleman presented with a one-day history of fever, chills, and altered mental status. His past medical history was significant for Type 2 Diabetes Mellitus that had been complicated by foot ulcers resulting in amputation of multiple toes, end stage renal disease requiring hemodialysis thrice a week, and congestive heart failure. He had a remote history of smoking 25 years ago and drank alcohol socially. He denied any recreational drug use.

**Figure 1:**
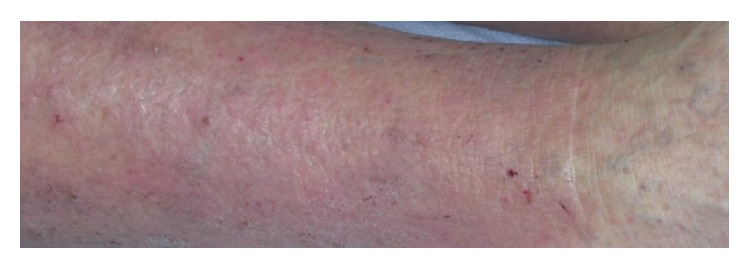
Changes of stasis dermatitis with skin breaks.

Vital signs on admission were as follows: temperature 102 F, heart rate 105 beats per minute, blood pressure 95/75 mm of Hg, and respiratory rate 15 breaths per minute. Examination revealed lower limb edema due to chronic venous stasis, changes of stasis dermatitis, and multiple superficial ulcerations (Figure 1). Multiple missing toes due to prior amputations secondary to diabetic ulcers were also noted.

Results from his laboratory workup were as follows: hemoglobin 11.4 gm/dL, leucocytes 8.3 K/*μ*L, and platelets 166 K/*μ*L. His creatinine was elevated at 4 mg/dL, which was expected with his end stage renal disease. Procalcitonin was noted to be 93 ng/mL (normal level < 0.05 ng/mL), and lactic acid was 2.1 mmol/L. Urinalysis did not show any abnormalities. On chest X-ray, lung fields appeared to be clear. Ultrasound of the lower extremities did not show any evidence of deep vein thrombosis. Blood cultures were drawn and in the meanwhile the patient was started on empiric antibiotics and fluids.

Patient was initially treated with clindamycin for possible cellulitis as the source of sepsis. During the second day of admission blood cultures grew* Pasteurella multocida*, a component of the normal upper respiratory tract flora of domestic animals. This triggered the medical team to ask about recent animal contact. The patient then admitted that his dogs had recently licked the wounds on his legs. Sensitivities showed a pan sensitive organism and the patient was switched to ciprofloxacin for two weeks. The patient followed with the infectious diseases clinic after completion of his antibiotics, repeat blood cultures at the time were negative, and the patient was doing well.

## 3. Discussion

*Pasteurella multocida* are small gram-negative coccobacilli, commonly found in oral microbiota of 60% of dogs and 70–90% of cats. Infections are usually transmitted to humans as a result of cat scratches and cat or dog bites but can also rarely be seen following licks from these animals [2]. Cat bites pose a greater risk of infection as they tend to produce deeper puncture wounds. Kissing animals has been reported as a source of infection in several cases [3]. A single case of probable human to human transmission has been reported, via close contact with colonised individuals [4]. However, infections have also been reported to occur without any apparent animal exposure [5]. In our case the patient's dogs licking his leg wounds was the probable source of infection. Our case highlights that* P. multocida* infection can occur even after casual contact with domestic animals and does not necessarily require an animal bite.

*Pasteurella* typically causes cellulitis or abscess formation following dog or cat bites. A large retrospective analysis by Escande and Lion [6] of 958 patients with* Pasteurella* infections showed that the most common form of infection was wound infection (66%), followed by respiratory infection in 19% and bacteremia in 11% of patients. Osteomyelitis can result from either local extension of soft tissue infection or from direct inoculation of the periosteum from cat or dog bite. Septic arthritis commonly occurs after cat and dog bites distal to the involved joint without direct inoculation of the joint [7]. There is a predilection for involvement of prosthetic joints or joints that have been damaged by degenerative joint disease. Respiratory infections like pneumonia, empyema, and lung abscess are seen in patient with underlying pulmonary diseases like chronic obstructive lung disease. Invasive* Pasteurella* infections like bacteremia remain relatively rare, but severe with high case fatality rate. Bacteremia in majority of the cases accompanies localised infection, most commonly cellulitis. Serious systemic infections usually occur in patient with chronic predisposing conditions (cirrhosis, chronic renal failure, diabetes mellitus, hematologic malignancies, or solid organ transplantation) [8]. Our patient had poorly controlled diabetes mellitus and end stage renal disease requiring hemodialysis, both of which predisposed him to develop bacteremia.

Ebright et al. conducted a large twenty-year review of all the cases of* Pasteurella* seen in Detroit Medical Centre. They noted that most infections were in children, although all the patients with bacteremia were adults with immunocompromising conditions (similar to our patient). Some patients denied any animal contact [9].

There are no clinical trials evaluating the efficacy of different antibiotics for treatment of* Pasteurella multocida* infections.* P. multocida* is usually susceptible to a number of antibiotics including penicillin, amoxicillin-clavulanate, piperacillin-tazobactam, fluoroquinolones (levofloxacin and ciprofloxacin), third and fourth generation cephalosporins (ceftriaxone, cefixime), and trimethoprim-sulphamethoxazole. The drug of choice for monomicrobial infections is penicillin. Penicillin resistance is infrequent but has been described [10]. Treatment failure is typically seen with vancomycin and clindamycin. Our patient was treated with ciprofloxacin for two weeks and made an uneventful recovery.

*P. multocida* infections when limited to the skin and soft tissues have a good prognosis. However, studies have shown a mortality rate ranging from 7 to 31% in patient with* P. multocida* bacteremia [9]. In conclusion,* P. multocida* infections are associated with a high rate of mortality in patient with serious comorbid conditions. The number of pets in urban households is on rise and this predisposes to increased exposure to animals and subsequently increase in the incidence of pet related infections such as* Pasteurella multocida*. It is important to maintain a high index of suspicion for this infection in patients with exposure to domestic animals and promptly initiate appropriate antibiotic therapy.
